# (2*E*)-1-(2,4-Dichloro­phen­yl)-3-[3-(4-nitro­phen­yl)-1-phenyl-1*H*-pyrazol-4-yl]prop-2-en-1-one

**DOI:** 10.1107/S1600536812003960

**Published:** 2012-02-04

**Authors:** Arun M. Isloor, Shridhar Malladi, Thomas Gerber, Benjamin van Brecht, Richard Betz

**Affiliations:** a Organic Electronics Division, Department of Chemistry, National Institute of Technology - Karnataka, Surathkal, Mangalore 575 025, India; b Nelson Mandela Metropolitan University, Summerstrand Campus, Department of Chemistry, University Way, Summerstrand, PO Box 77000, Port Elizabeth 6031, South Africa

## Abstract

In the title compound, C_24_H_15_Cl_2_N_3_O_3_, the C=C double bond is *E* configured. The 1-phenyl-1*H*-pyrazole moiety is roughly planar (r.m.s. deviation of all fitted non-H atoms = 0.0780 Å), but the mean planes of the two components are inclined at an angle of 9.95 (7)°. The mean plane defined by the non-H atoms of the 1*H*-pyrazole ring encloses angles of 9.95 (7), 24.54 (6) and 43.02 (6)° with the mean planes of the different benzene rings. In the crystal, C—H⋯O contacts are present and result in the formation of a double-layer two-dimensional network lying parallel to (110). The shortest inter­centroid distance between two aromatic systems is 3.5455 (7) Å and is apparent between two pyrazole systems. Further π–π inter­actions are manifest between a pair of 4-nitro­phenyl rings [centroid-to-centroid distance = 3.6443 (7) Å] and a pair of 2,4-dichloro­phenyl rings [centroid-to-centroid distance = 3.7797 (7) Å].

## Related literature

For general background on the pharmaceutical and biological activity of pyrazole compounds, see: Isloor *et al.* (2009 ▶); Vijesh *et al.* (2010 ▶); Sharma *et al.* (2010 ▶); Rostom *et al.* (2003 ▶); Ghorab *et al.* (2010 ▶); Amnekar & Bhusari (2010 ▶). For graph-set analysis of hydrogen bonds, see: Etter *et al.* (1990 ▶); Bernstein *et al.* (1995 ▶).
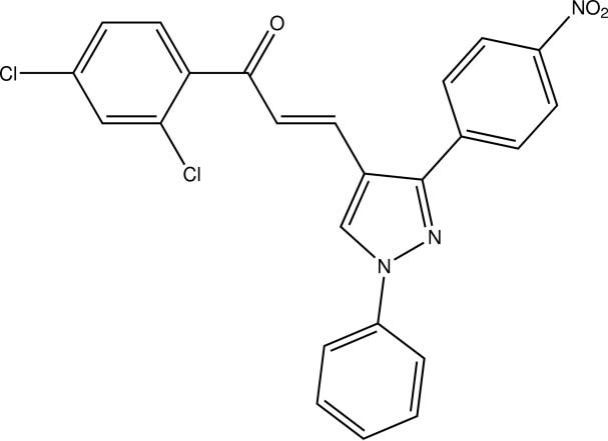


## Experimental

### 

#### Crystal data

C_24_H_15_Cl_2_N_3_O_3_
*M* *_r_* = 464.29
Triclinic, 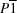

*a* = 8.3343 (3) Å
*b* = 9.3115 (4) Å
*c* = 13.8699 (6) Å
α = 92.896 (2)°
β = 104.669 (2)°
γ = 96.060 (2)°
*V* = 1032.12 (7) Å^3^
*Z* = 2
Mo *K*α radiation
μ = 0.35 mm^−1^
*T* = 200 K
0.53 × 0.30 × 0.13 mm

#### Data collection

Bruker APEXII CCD diffractometer
Absorption correction: multi-scan (*SADABS*; Bruker, 2008 ▶) *T*_min_ = 0.931, *T*_max_ = 1.000
18344 measured reflections
5116 independent reflections
4588 reflections with *I* > 2σ(*I*)
*R*_int_ = 0.013

#### Refinement

*R*[*F* ^2^ > 2σ(*F* ^2^)] = 0.030
*wR*(*F* ^2^) = 0.086
*S* = 1.02
5116 reflections
304 parameters
H-atom parameters constrained
Δρ_max_ = 0.36 e Å^−3^
Δρ_min_ = −0.21 e Å^−3^

### 

Data collection: *APEX2* (Bruker, 2010 ▶); cell refinement: *SAINT* (Bruker, 2010 ▶); data reduction: *SAINT*; program(s) used to solve structure: *SHELXS97* (Sheldrick, 2008 ▶); program(s) used to refine structure: *SHELXL97* (Sheldrick, 2008 ▶); molecular graphics: *ORTEP-3* (Farrugia, 1997 ▶) and *Mercury* (Macrae *et al.*, 2008 ▶); software used to prepare material for publication: *SHELXL97* and *PLATON* (Spek, 2009 ▶).

## Supplementary Material

Crystal structure: contains datablock(s) I, global. DOI: 10.1107/S1600536812003960/su2371sup1.cif

Supplementary material file. DOI: 10.1107/S1600536812003960/su2371Isup2.cdx

Structure factors: contains datablock(s) I. DOI: 10.1107/S1600536812003960/su2371Isup3.hkl

Supplementary material file. DOI: 10.1107/S1600536812003960/su2371Isup4.cml

Additional supplementary materials:  crystallographic information; 3D view; checkCIF report

## Figures and Tables

**Table 1:** Hydrogen-bond geometry (Å, °)

*D*—H⋯*A*	*D*—H	H⋯*A*	*D*⋯*A*	*D*—H⋯*A*
C5—H5⋯O1^i^	0.95	2.39	3.3421 (14)	176
C36—H36⋯O3^ii^	0.95	2.41	3.3139 (15)	160

Symmetry codes: (i) 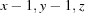; (ii) 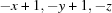.

**Fig. 1.**
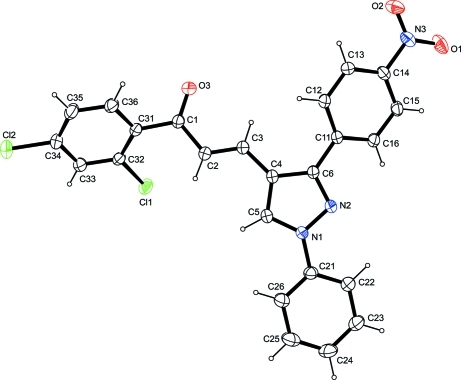
The molecular structure of the title compound, with atom labels and displacement ellipsoids drawn at the 50% probability level.

**Fig. 2.**
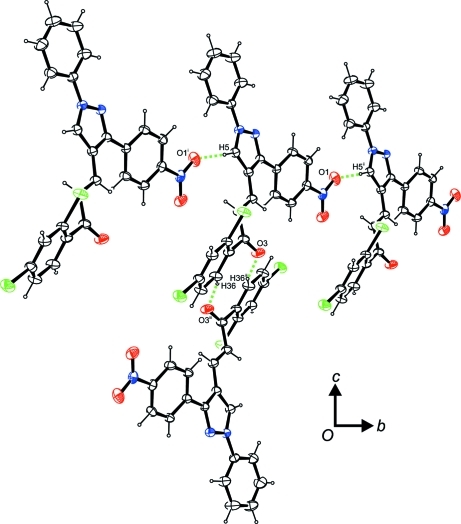
A partial view along the a axis of the crystal packing of the title compound, showing the C—H···O intermolecular contacts [Symmetry operators: (i) x - 1, y - 1, z; (ii) x + 1, y + 1, z; (iii) -x + 1, -y + 1, -z].

**Fig. 3.**
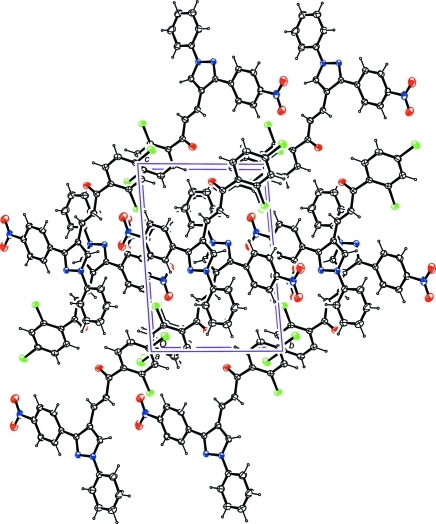
A view along the a axis of the crystal packing of the title compound (displacement ellipsoids are drawn at 50% probability level).

## supplementary crystallographic information

### Comment

The pyrazole ring is an important structural motif found in several pharmaceutically active compounds. Because of its easy preparation and rich biological activity, the pyrazole skeleton plays an important role in biologically active compounds such as antibacterial (Isloor *et al.*, 2009; Vijesh *et al.*, 2010), anti-inflammatory (Sharma *et al.*, 2010), analgesic (Rostom *et al.*, 2003), anticancer, radioprotective (Ghorab *et al.*, 2010) and anti-convulsant agents (Amnekar & Bhusari, 2010). Prompted by the diverse activities of pyrazole derivatives, we have synthesized the title compound to study its crystal structure.

In the title compound the C═C double bond in the Michael system adopts (*E*)-configuration (Fig. 1). The 1-phenyl-1*H*-pyrazole moiety is essentially planar (r.m.s. deviation of all fitted non-hydrogen atoms = 0.0780 Å). However, the mean planes of the two components are inclined at an angle of 9.95 (7)°.

The *N*-bonded phenyl ring B (C21–C26), the 4-nitrophenyl ring C (C11–C16), and the 2,4-dichlorophenyl ring D (C31–C36) are inclined to the mean plane of the central heterocyclic five-membered ring A (N1,N2,C4–C6) by 9.95 (7), 24.54 (6) and 43.06 (6) °, respectively. The mean planes defined the phenyl rings (B, C and D) are inclined to one another by angles of B/C = 16.28 (6)°, C/D = 28.40 (6)° and B/D = 40.14 (6)°.

In the crystal, C—H···O contacts whose range falls by more than 0.3 Å below the sum of van der Waals radii of the corresponding atoms are present. They are supported by one of the H atoms of the pyrazole system on the one hand and one of the H atoms on the dichlorophenyl moiety on the other hand. While the former of these contacts applies exclusively to one of the O atoms (O1) on the nitro group as acceptor, the latter ones are apparent in conjunction with the O atom (O3) on the Michael system (Table 1 and Fig. 2). In terms of graph-set analysis (Etter *et al.*, 1990; Bernstein *et al.*, 1995), the descriptor for the C—H···O contacts is *C*^1^_1_(11)*R*^2^_2_(10) on the unitary level.

The shortest intercentroid distance between two aromatic systems is 3.5455 (7) Å involving inversion related pyrazole systems [CgA···CgA^i^]. Further π–π interactions are manifest between inversion related 4-nitrophenyl rings (CgC···CgC^ii^ = 3.6443 (7) Å) and inversion related 2,4-dichlorophenyl rings (CgD···CgD^iii^ = 3.7797 (7) Å) [symmetry codes: (i) -*x* + 2, -*y* + 1, -*z* + 1; (ii) -*x* + 2, -*y* + 2, -*z* + 1; (iii) -*x* + 1, -*y*, -*z*].

In total, the molecules are connected into a double layer two-dimensional network lying parallel to plane (110) [Fig. 3].

### Experimental

To a cold, stirred mixture of methanol (20 ml) and sodium hydroxide (12.09 mmol) was added 2,4-dichloroacetophenone (4.03 mmol). The reaction mixture was stirred for 10 min. To this was added 3-(4-nitrophenyl)-1-phenyl-1*H*-pyrazole-4-carbaldehyde (4.03 mmol) followed by tetrahydrofuran (30 ml). The solution was further stirred at 0°C for 2 h and then at room temperature for 5 h. It was then poured into ice cold water. The resulting solution was neutralized with diluted hydrochloric acid. The solid that separated was filtered, washed with water, dried and crystallized from ethanol. Yield: 1.48 g, 79.39% (m.p. 478–480 K).

### Refinement

Carbon-bound H atoms were placed in calculated positions (C—H 0.95 Å) and were included in the refinement in the riding model approximation, with *U*(H) = 1.2*U*_eq_(C).

### Crystal data

**Table:**

C_24_H_15_Cl_2_N_3_O_3_	*Z* = 2
*M**_r_* = 464.29	*F*(000) = 476
Triclinic, *P*1	*D*_x_ = 1.494 Mg m^−^^3^
Hall symbol: -P 1	Melting point = 478–480 K
*a* = 8.3343 (3) Å	Mo *K*α radiation, λ = 0.71073 Å
*b* = 9.3115 (4) Å	Cell parameters from 9938 reflections
*c* = 13.8699 (6) Å	θ = 2.6–28.3°
α = 92.896 (2)°	µ = 0.35 mm^−^^1^
β = 104.669 (2)°	*T* = 200 K
γ = 96.060 (2)°	Plate, yellow
*V* = 1032.12 (7) Å^3^	0.53 × 0.30 × 0.13 mm

### Data collection

**Table:**

Bruker APEXII CCD diffractometer	5116 independent reflections
Radiation source: fine-focus sealed tube	4588 reflections with *I* > 2σ(*I*)
Graphite monochromator	*R*_int_ = 0.013
φ and ω scans	θ_max_ = 28.4°, θ_min_ = 1.5°
Absorption correction: multi-scan (*SADABS*; Bruker, 2008)	*h* = −11→11
*T*_min_ = 0.931, *T*_max_ = 1.000	*k* = −11→12
18344 measured reflections	*l* = −18→18

### Refinement

**Table:**

Refinement on *F*^2^	Primary atom site location: structure-invariant direct methods
Least-squares matrix: full	Secondary atom site location: difference Fourier map
*R*[*F*^2^ > 2σ(*F*^2^)] = 0.030	Hydrogen site location: inferred from neighbouring sites
*wR*(*F*^2^) = 0.086	H-atom parameters constrained
*S* = 1.02	*w* = 1/[σ^2^(*F*_o_^2^) + (0.046*P*)^2^ + 0.3579*P*] where *P* = (*F*_o_^2^ + 2*F*_c_^2^)/3
5116 reflections	(Δ/σ)_max_ = 0.001
304 parameters	Δρ_max_ = 0.36 e Å^−^^3^
0 restraints	Δρ_min_ = −0.21 e Å^−^^3^

### Fractional atomic coordinates and isotropic or equivalent isotropic displacement parameters (Å^2^)

**Table:**

	*x*	*y*	*z*	*U*_iso_*/*U*_eq_	
Cl1	0.61554 (4)	0.04973 (3)	0.23425 (2)	0.03315 (9)	
Cl2	0.09111 (4)	−0.11303 (4)	−0.07809 (2)	0.03888 (9)	
O1	1.42080 (12)	1.17926 (10)	0.43746 (8)	0.0407 (2)	
O2	1.27245 (13)	1.14934 (11)	0.28397 (7)	0.0398 (2)	
O3	0.69233 (12)	0.43425 (10)	0.10434 (6)	0.0350 (2)	
N1	0.78730 (12)	0.53362 (9)	0.57632 (7)	0.02186 (18)	
N2	0.89358 (12)	0.65279 (10)	0.57207 (7)	0.02233 (18)	
N3	1.30319 (13)	1.11802 (11)	0.37064 (8)	0.0286 (2)	
C1	0.61545 (15)	0.35986 (12)	0.15177 (8)	0.0248 (2)	
C2	0.63269 (15)	0.39188 (12)	0.25920 (8)	0.0256 (2)	
H2	0.5617	0.3378	0.2917	0.029 (4)*	
C3	0.74872 (14)	0.49747 (12)	0.31106 (8)	0.0238 (2)	
H3	0.8177	0.5483	0.2758	0.030 (4)*	
C4	0.77783 (13)	0.54064 (11)	0.41643 (8)	0.0219 (2)	
C5	0.71502 (14)	0.46538 (11)	0.48506 (8)	0.0233 (2)	
H5	0.6355	0.3811	0.4706	0.034 (4)*	
C6	0.88826 (13)	0.65830 (11)	0.47531 (8)	0.0205 (2)	
C11	0.99209 (13)	0.77692 (11)	0.44612 (8)	0.0206 (2)	
C12	0.95250 (15)	0.82678 (12)	0.35047 (8)	0.0261 (2)	
H12	0.8545	0.7837	0.3022	0.035 (4)*	
C13	1.05435 (15)	0.93829 (12)	0.32506 (8)	0.0271 (2)	
H13	1.0283	0.9710	0.2597	0.039 (4)*	
C14	1.19461 (13)	1.00064 (11)	0.39712 (8)	0.0236 (2)	
C15	1.23561 (14)	0.95718 (12)	0.49328 (8)	0.0252 (2)	
H15	1.3312	1.0037	0.5419	0.038 (4)*	
C16	1.13429 (14)	0.84452 (12)	0.51712 (8)	0.0239 (2)	
H16	1.1616	0.8126	0.5826	0.030 (4)*	
C21	0.77030 (14)	0.49032 (12)	0.67088 (8)	0.0240 (2)	
C22	0.87694 (18)	0.55949 (14)	0.75788 (9)	0.0331 (3)	
H22	0.9608	0.6352	0.7548	0.043 (4)*	
C23	0.8601 (2)	0.51708 (15)	0.84967 (10)	0.0414 (3)	
H23	0.9330	0.5642	0.9096	0.054 (5)*	
C24	0.7381 (2)	0.40681 (15)	0.85476 (10)	0.0414 (3)	
H24	0.7266	0.3784	0.9177	0.054 (5)*	
C25	0.63317 (19)	0.33854 (16)	0.76723 (11)	0.0403 (3)	
H25	0.5493	0.2629	0.7705	0.056 (5)*	
C26	0.64836 (16)	0.37876 (14)	0.67454 (9)	0.0322 (3)	
H26	0.5764	0.3307	0.6147	0.046 (5)*	
C31	0.48885 (14)	0.23647 (12)	0.09753 (8)	0.0237 (2)	
C32	0.47465 (14)	0.09718 (12)	0.12861 (8)	0.0240 (2)	
C33	0.35401 (15)	−0.01128 (12)	0.07491 (8)	0.0271 (2)	
H33	0.3472	−0.1063	0.0968	0.040 (4)*	
C34	0.24377 (15)	0.02196 (13)	−0.01124 (8)	0.0277 (2)	
C35	0.25331 (16)	0.15887 (14)	−0.04498 (9)	0.0315 (3)	
H35	0.1762	0.1799	−0.1043	0.045 (4)*	
C36	0.37677 (16)	0.26473 (13)	0.00889 (8)	0.0292 (2)	
H36	0.3857	0.3585	−0.0147	0.034 (4)*	

### Atomic displacement parameters (Å^2^)

**Table:**

	*U*^11^	*U*^22^	*U*^33^	*U*^12^	*U*^13^	*U*^23^
Cl1	0.04021 (17)	0.02982 (15)	0.02282 (14)	0.00229 (11)	−0.00347 (11)	0.00416 (10)
Cl2	0.03790 (17)	0.03595 (17)	0.03315 (16)	−0.01017 (13)	−0.00076 (12)	−0.00578 (12)
O1	0.0281 (4)	0.0329 (5)	0.0548 (6)	−0.0099 (4)	0.0046 (4)	0.0044 (4)
O2	0.0452 (5)	0.0380 (5)	0.0402 (5)	−0.0023 (4)	0.0201 (4)	0.0115 (4)
O3	0.0458 (5)	0.0326 (4)	0.0242 (4)	−0.0089 (4)	0.0100 (4)	0.0028 (3)
N1	0.0256 (4)	0.0183 (4)	0.0212 (4)	−0.0012 (3)	0.0069 (3)	0.0010 (3)
N2	0.0257 (4)	0.0182 (4)	0.0214 (4)	−0.0023 (3)	0.0052 (3)	0.0006 (3)
N3	0.0259 (5)	0.0227 (4)	0.0393 (6)	0.0001 (4)	0.0131 (4)	0.0039 (4)
C1	0.0310 (5)	0.0209 (5)	0.0199 (5)	−0.0009 (4)	0.0040 (4)	−0.0003 (4)
C2	0.0306 (5)	0.0241 (5)	0.0205 (5)	−0.0032 (4)	0.0067 (4)	−0.0015 (4)
C3	0.0284 (5)	0.0207 (5)	0.0211 (5)	−0.0016 (4)	0.0064 (4)	−0.0006 (4)
C4	0.0232 (5)	0.0192 (5)	0.0215 (5)	−0.0013 (4)	0.0045 (4)	−0.0009 (4)
C5	0.0254 (5)	0.0195 (5)	0.0232 (5)	−0.0020 (4)	0.0055 (4)	−0.0010 (4)
C6	0.0219 (5)	0.0183 (4)	0.0198 (5)	0.0003 (4)	0.0040 (4)	0.0000 (4)
C11	0.0225 (5)	0.0175 (4)	0.0205 (5)	−0.0005 (4)	0.0044 (4)	−0.0003 (4)
C12	0.0281 (5)	0.0246 (5)	0.0204 (5)	−0.0048 (4)	0.0006 (4)	0.0010 (4)
C13	0.0322 (6)	0.0259 (5)	0.0207 (5)	−0.0029 (4)	0.0046 (4)	0.0039 (4)
C14	0.0232 (5)	0.0191 (5)	0.0288 (5)	−0.0010 (4)	0.0091 (4)	0.0016 (4)
C15	0.0231 (5)	0.0222 (5)	0.0263 (5)	−0.0016 (4)	0.0010 (4)	−0.0004 (4)
C16	0.0260 (5)	0.0223 (5)	0.0201 (5)	−0.0004 (4)	0.0012 (4)	0.0008 (4)
C21	0.0295 (5)	0.0216 (5)	0.0222 (5)	0.0038 (4)	0.0087 (4)	0.0041 (4)
C22	0.0443 (7)	0.0281 (6)	0.0249 (6)	−0.0043 (5)	0.0092 (5)	0.0008 (4)
C23	0.0618 (9)	0.0359 (7)	0.0228 (6)	−0.0046 (6)	0.0087 (6)	0.0018 (5)
C24	0.0604 (9)	0.0387 (7)	0.0279 (6)	0.0023 (6)	0.0167 (6)	0.0108 (5)
C25	0.0454 (8)	0.0393 (7)	0.0369 (7)	−0.0044 (6)	0.0135 (6)	0.0149 (6)
C26	0.0349 (6)	0.0311 (6)	0.0283 (6)	−0.0030 (5)	0.0062 (5)	0.0073 (5)
C31	0.0304 (5)	0.0217 (5)	0.0169 (5)	−0.0009 (4)	0.0048 (4)	−0.0010 (4)
C32	0.0290 (5)	0.0245 (5)	0.0165 (4)	0.0015 (4)	0.0030 (4)	0.0011 (4)
C33	0.0332 (6)	0.0223 (5)	0.0233 (5)	−0.0012 (4)	0.0050 (4)	0.0012 (4)
C34	0.0291 (5)	0.0274 (5)	0.0225 (5)	−0.0026 (4)	0.0032 (4)	−0.0042 (4)
C35	0.0361 (6)	0.0319 (6)	0.0205 (5)	0.0018 (5)	−0.0023 (4)	0.0009 (4)
C36	0.0398 (6)	0.0233 (5)	0.0209 (5)	0.0013 (5)	0.0022 (5)	0.0030 (4)

### Geometric parameters (Å, º)

**Table:**

Cl1—C32	1.7391 (11)	C13—H13	0.9500
Cl2—C34	1.7336 (11)	C14—C15	1.3829 (16)
O1—N3	1.2302 (14)	C15—C16	1.3829 (15)
O2—N3	1.2210 (14)	C15—H15	0.9500
O3—C1	1.2203 (14)	C16—H16	0.9500
N1—C5	1.3493 (14)	C21—C22	1.3850 (16)
N1—N2	1.3582 (12)	C21—C26	1.3868 (16)
N1—C21	1.4277 (13)	C22—C23	1.3878 (17)
N2—C6	1.3354 (14)	C22—H22	0.9500
N3—C14	1.4657 (14)	C23—C24	1.384 (2)
C1—C2	1.4722 (15)	C23—H23	0.9500
C1—C31	1.5014 (15)	C24—C25	1.382 (2)
C2—C3	1.3397 (15)	C24—H24	0.9500
C2—H2	0.9500	C25—C26	1.3892 (17)
C3—C4	1.4481 (14)	C25—H25	0.9500
C3—H3	0.9500	C26—H26	0.9500
C4—C5	1.3832 (15)	C31—C32	1.3899 (15)
C4—C6	1.4248 (14)	C31—C36	1.3986 (16)
C5—H5	0.9500	C32—C33	1.3842 (15)
C6—C11	1.4675 (14)	C33—C34	1.3817 (16)
C11—C12	1.3990 (15)	C33—H33	0.9500
C11—C16	1.3998 (14)	C34—C35	1.3815 (17)
C12—C13	1.3875 (15)	C35—C36	1.3823 (16)
C12—H12	0.9500	C35—H35	0.9500
C13—C14	1.3815 (16)	C36—H36	0.9500
			
C5—N1—N2	112.12 (9)	C15—C16—C11	120.93 (10)
C5—N1—C21	127.88 (9)	C15—C16—H16	119.5
N2—N1—C21	119.91 (9)	C11—C16—H16	119.5
C6—N2—N1	105.11 (8)	C22—C21—C26	120.80 (11)
O2—N3—O1	123.73 (10)	C22—C21—N1	119.53 (10)
O2—N3—C14	118.48 (10)	C26—C21—N1	119.66 (10)
O1—N3—C14	117.79 (10)	C21—C22—C23	119.37 (12)
O3—C1—C2	122.78 (10)	C21—C22—H22	120.3
O3—C1—C31	118.98 (10)	C23—C22—H22	120.3
C2—C1—C31	118.10 (10)	C24—C23—C22	120.62 (13)
C3—C2—C1	119.88 (10)	C24—C23—H23	119.7
C3—C2—H2	120.1	C22—C23—H23	119.7
C1—C2—H2	120.1	C25—C24—C23	119.27 (12)
C2—C3—C4	125.46 (10)	C25—C24—H24	120.4
C2—C3—H3	117.3	C23—C24—H24	120.4
C4—C3—H3	117.3	C24—C25—C26	121.08 (12)
C5—C4—C6	104.13 (9)	C24—C25—H25	119.5
C5—C4—C3	126.45 (10)	C26—C25—H25	119.5
C6—C4—C3	129.11 (10)	C21—C26—C25	118.86 (12)
N1—C5—C4	107.48 (9)	C21—C26—H26	120.6
N1—C5—H5	126.3	C25—C26—H26	120.6
C4—C5—H5	126.3	C32—C31—C36	117.80 (10)
N2—C6—C4	111.14 (9)	C32—C31—C1	125.11 (10)
N2—C6—C11	118.25 (9)	C36—C31—C1	117.09 (10)
C4—C6—C11	130.60 (9)	C33—C32—C31	121.95 (10)
C12—C11—C16	118.75 (10)	C33—C32—Cl1	117.05 (9)
C12—C11—C6	122.43 (9)	C31—C32—Cl1	120.93 (8)
C16—C11—C6	118.82 (9)	C34—C33—C32	118.38 (10)
C13—C12—C11	120.93 (10)	C34—C33—H33	120.8
C13—C12—H12	119.5	C32—C33—H33	120.8
C11—C12—H12	119.5	C35—C34—C33	121.64 (11)
C14—C13—C12	118.35 (10)	C35—C34—Cl2	119.80 (9)
C14—C13—H13	120.8	C33—C34—Cl2	118.57 (9)
C12—C13—H13	120.8	C34—C35—C36	118.94 (11)
C13—C14—C15	122.50 (10)	C34—C35—H35	120.5
C13—C14—N3	118.63 (10)	C36—C35—H35	120.5
C15—C14—N3	118.87 (10)	C35—C36—C31	121.26 (11)
C14—C15—C16	118.51 (10)	C35—C36—H36	119.4
C14—C15—H15	120.7	C31—C36—H36	119.4
C16—C15—H15	120.7		
			
C5—N1—N2—C6	−0.46 (12)	C14—C15—C16—C11	−0.82 (17)
C21—N1—N2—C6	176.30 (9)	C12—C11—C16—C15	−0.90 (17)
O3—C1—C2—C3	−6.97 (19)	C6—C11—C16—C15	−179.72 (10)
C31—C1—C2—C3	177.41 (11)	C5—N1—C21—C22	168.36 (12)
C1—C2—C3—C4	179.47 (11)	N2—N1—C21—C22	−7.84 (16)
C2—C3—C4—C5	12.94 (19)	C5—N1—C21—C26	−11.06 (17)
C2—C3—C4—C6	−174.38 (12)	N2—N1—C21—C26	172.74 (10)
N2—N1—C5—C4	1.10 (13)	C26—C21—C22—C23	−0.6 (2)
C21—N1—C5—C4	−175.35 (10)	N1—C21—C22—C23	−179.98 (12)
C6—C4—C5—N1	−1.21 (12)	C21—C22—C23—C24	0.0 (2)
C3—C4—C5—N1	172.94 (10)	C22—C23—C24—C25	0.3 (2)
N1—N2—C6—C4	−0.35 (12)	C23—C24—C25—C26	0.0 (2)
N1—N2—C6—C11	−179.79 (9)	C22—C21—C26—C25	0.87 (19)
C5—C4—C6—N2	0.98 (12)	N1—C21—C26—C25	−179.71 (12)
C3—C4—C6—N2	−172.95 (11)	C24—C25—C26—C21	−0.6 (2)
C5—C4—C6—C11	−179.66 (11)	O3—C1—C31—C32	133.57 (13)
C3—C4—C6—C11	6.41 (19)	C2—C1—C31—C32	−50.63 (16)
N2—C6—C11—C12	−154.77 (11)	O3—C1—C31—C36	−46.48 (16)
C4—C6—C11—C12	25.91 (18)	C2—C1—C31—C36	129.32 (12)
N2—C6—C11—C16	24.00 (15)	C36—C31—C32—C33	−0.28 (17)
C4—C6—C11—C16	−155.32 (11)	C1—C31—C32—C33	179.67 (11)
C16—C11—C12—C13	1.85 (17)	C36—C31—C32—Cl1	176.56 (9)
C6—C11—C12—C13	−179.37 (11)	C1—C31—C32—Cl1	−3.49 (16)
C11—C12—C13—C14	−1.05 (18)	C31—C32—C33—C34	−0.93 (18)
C12—C13—C14—C15	−0.76 (18)	Cl1—C32—C33—C34	−177.88 (9)
C12—C13—C14—N3	179.71 (10)	C32—C33—C34—C35	0.97 (18)
O2—N3—C14—C13	−5.21 (16)	C32—C33—C34—Cl2	−179.36 (9)
O1—N3—C14—C13	174.93 (11)	C33—C34—C35—C36	0.21 (19)
O2—N3—C14—C15	175.23 (11)	Cl2—C34—C35—C36	−179.46 (10)
O1—N3—C14—C15	−4.62 (16)	C34—C35—C36—C31	−1.5 (2)
C13—C14—C15—C16	1.68 (17)	C32—C31—C36—C35	1.50 (18)
N3—C14—C15—C16	−178.78 (10)	C1—C31—C36—C35	−178.46 (11)

### Hydrogen-bond geometry (Å, º)

**Table:**

*D*—H···*A*	*D*—H	H···*A*	*D*···*A*	*D*—H···*A*
C5—H5···O1^i^	0.95	2.39	3.3421 (14)	176
C36—H36···O3^ii^	0.95	2.41	3.3139 (15)	160

Symmetry codes: (i) *x*−1, *y*−1, *z*; (ii) −*x*+1, −*y*+1, −*z*.
